# Chloral in Hooping-Cough and Asthma

**Published:** 1874-03

**Authors:** 


CHLORAL IN HOOPING-COUGH AND ASTHMA.—Dr. R. L. Moore (*Med. & Surg. Reporter*) says: We assert, that chloral is the best known remedy for hooping-cough in the whole materia medica. Our favorite formula is as follows:

℞—Chloral hydrat., ʒiv; aquæ puræ fl.ʒss; syr. simp., fl.ʒiss; ol. gaultheriæ, gtt. vj. M. This we keep constantly prepared, and never knew it to fail us from long keeping. Upon the accession of a paroxysm of asthma a full dose is to be taken, which often cuts it short; further treatment to be governed by circumstances. In hooping-cough we order from fifteen drops up to the adult dose, every four or five hours, according to age. We direct the remedy to be given steadily for a number of days, increasing the doses at night, and finally push it until they sleep after every dose, if the cough is not moderated or arrested. And we claim that it *will* arrest it, cut it short, abort it, in a large majority of cases. These are "empirical facts" gathered from a tolerably large experience. We instruct those using the above never to dilute it with water when about to take a dose, but with syrup. Water seems to develop the bad taste of chloral, which is its great drawback.

